# Depression in older Turkish immigrants and natives in Germany: a comparative analysis of risk and protective factors

**DOI:** 10.1186/s12889-025-24954-9

**Published:** 2025-10-31

**Authors:** N. Tugba Bahar, Jasmin Tahmaseb-McConatha, Frieder R. Lang

**Affiliations:** 1 Institute of Psychogerontology, Friedrich-Alexander University Erlangen-Nürnberg, Kobergerstr. 62, Nuremberg, 90408 Germany; 2 Department of Health Care Services, Vocational High School of Health Services, Giresun University, Giresun, 28049 Turkey; 3 Department of Psychology, West Chester University of Pennsylvania, West Chester, PA 19383 USA

**Keywords:** Gender differences, Ethnicity, Depression, Social support, Subjective health

## Abstract

**Background:**

The purpose of this study was to explore the risk and protective factors for depression in the context of gender and ethnicity, with a particular focus on the role of social relationships and subjective health, by comparing older Turkish immigrants living in Germany with a matched sample of German nonimmigrant older adults with similar sociodemographic characteristics.

**Methods:**

The study participants included 195 Turkish immigrants and 195 older German natives (75+). Participants were administered questionnaires on depression, subjective health, and social relationships.

**Results:**

Ethnicity, subjective health and perceived social support were the main predictors of depression: Turkish immigrants and women scored higher levels of depressive symptoms than their nonimmigrant and male counterparts. High levels of satisfaction with friendships, perceived social support, and subjective health were found to play a protective role against depressive symptoms in both groups. Family satisfaction moderated the relationship between ethnicity and depression: low family satisfaction was associated with a greater risk of depression in the Turkish sample but not in the German sample. Received social support also moderated the effect of gender and ethnicity on depression. Receiving greater support was related to a higher risk of depression in Turkish and German women, but to a lesser degree in Turkish men and was not observed in German men.

**Conclusion:**

The findings indicated that appraisals and mental health effects of receiving support differed by both ethnicity and gender. Family satisfaction varied by ethnicity and played a moderating role in depression. Future research may focus on the basis of differences in depression in old age.

**Supplementary Information:**

The online version contains supplementary material available at 10.1186/s12889-025-24954-9.

## Background

Depression is a global health concern and one of the leading causes of disability, particularly in later life. It is characterized primarily by low mood, a sense of worthlessness, and withdrawal from social life [1, 2]. Late-life depression is particularly problematic as it is often undiagnosed or not treated appropriately. When left untreated it can lead to disability, decreased physical, cognitive, and social functioning, suicide, even premature mortality [3]. According to the World Health Organization [4] a minimum of 5% of the global population struggles with depression. The percentages are higher for older adults and at-risk marginalized populations [3]. The intersecting factors associated with an increased risk for depression include poor health [5], a history of immigration [6], being female, age, lower income and education, loss of a partner, living alone [7], and a lack of satisfying social support [8].

Although a global concern, studies have shown that depression rates, symptoms, and expression patterns vary by age, ethnicity, and culture. For example, depression rates are higher in Germany than in other European countries, with approximately 9.2% of the population struggling with depression symptoms [9]. Rates increase further with age, and for women over 75 years of age, rates are as high as 26.9% [10]. These percentages highlight the fact that older adults, particularly older women, are vulnerable for late-life depression and its associated concerns [6]. Later life vulnerabilities, losses, and stressful life events may challenge lifelong coping and self-regulation strategies, placing aging adults at increased risk for depression [3].

In addition to age-related challenges, there are migration-specific factors that uniquely shape the aging processes of immigrants. These factors include the pre-immigration experiences in the country of origin, difficulties faced during the immigration process, and structural barriers encountered post-immigration. Such immigration-specific multiple stressors effect health behaviors, mortality and morbidity risk, and ultimately both the physical and mental health status of older immigrants [11]. As immigrants age, they may face cumulative disadvantages, including acculturation stress, poverty, language competence, and a lack of support and services, that compound their vulnerability to poor health outcomes [12, 13]. While factors like acculturation stress and language barriers often begin earlier in the immigration process, they may intensify in later life due to cognitive decline, social role loss, and greater dependence on healthcare systems [11]. Research indicates that health inequalities between immigrant and native populations tend to widen with age, and that older immigrants often encounter barriers to accessing healthcare services [11, 14]. In this context, older immigrants are more vulnerable to mental health challenges due to the intersection of aging-related stressors and immigration-related challenges. As people live longer lives and an increasing number of immigrants age in host countries, it is necessary to explore the factors associated with the mental health of aging immigrants, particularly depression. Studies indicate that, globally, depression is one of the primary mental health concerns of later life, leading to a decline in overall well-being [12, 15].

### Aging immigrants in Germany

Global immigration is at an all-time high; consequently, an increasing number of immigrants are aging in host countries [11]. Germany has a large Turkish immigrant population of almost 2 million, accounting for 3.5% of the general population. This number, as defined by German Federal Statistical Office includes all individuals who have migrated to Germany since 1960s [16]. Turkish immigrants first arrived in the 1960 s and 1970 s as “*guest workers”.* Initially, these guest workers intended to return to Turkey with savings they accrued while working in Germany. However, circumstances often intervene, compelling many to remain in Germany as long-term immigrants. This liminal state can pose a considerable stress threat, particularly in later life, leading to mental health challenges [17, 18]. Moreover, most workers who immigrated to Germany during this period have lived and worked in Germany for many years and have reached the retirement age (65 or older). Germany is only now beginning to witness the aging of this so-called first generation of *guest workers*, primarily from Turkey, who arrived as part of post-war labor agreements [11]. In this vein, given that a significant number of older Turkish immigrants currently live in Germany, studies exploring this population’s mental health needs are necessar*y* and helpful in the development of services and programs that can address their overall well-being and improve the quality of their later years.

Research has indicated that immigrant/native disparities exist in later-life well-being, particularly in depression rates. In Europe, for example, older immigrants from non-European countries have been found to have lower levels of overall well-being than their native counterparts [19]. One crucial factor appears to be the socioeconomic advantages or disadvantages of the host country compared with the culture of origin [15]. To identify challenges faced by older immigrants, comparative studies with nonimmigrants of similar socioeconomic status (e.g., education, income, family status) are needed.

### Gender differences in depression

Studies have shown that depression rates are disproportionately higher for women [7], particularly for at-risk populations such as older immigrant women [6]. Across the life course, women face greater physical and mental health challenges [7, 9]. Gender stratification theory [20], which addresses gender-based roles and inequalities in power and privilege, postulates that, from early in life, girls and women tend to be accorded a lower status than men, leading to a lifetime of unequal access to health-promoting resources. This theory applies across cultural contexts, including both native and immigrant women. For example, large-scale comparative studies across 23 countries have shown that women report higher levels of depression than men do in all countries [21]. However, immigration to a new country may not always mitigate these inequalities and, immigrant women may face additional layers of disadvantage, in some cases, due to language barriers, social isolation, or dependency on family members. Similarly, recent research on immigrant populations in Europe highlighted that older immigrant women are more vulnerable to depression symptoms compared to their male counterparts [7]. Gender-based risk factors associated with cumulative disadvantage, including the feminization of poverty, education and income inequality, social norms and behaviors, gender-based discrimination and violence, and greater stress, are intersecting factors that negatively influence the well-being and subjective health of older women [6]. Exploring risk and protective factors among older immigrants, particularly immigrant women, is crucial.

### Subjective health and depression

Health challenges of late life (e.g., frailty, loneliness, isolation) are known to be related to increased rates of depression [22]. Self-assessments of physical health, in particular, tend to be predictive of mental health [5]. Subjective health is generally correlated with objective health, but it is also more complicated and influenced by personal and cultural factors such as resilience, personality, support, and socioeconomic resources [23]. In this vein, poor subjective health has been observed to be associated with increased risks of functional disabilities, chronic diseases, and the need for care [22]. Given that subjective health appears to play a powerful role in vulnerability to depression, one of the aims of this study is to measure the effect of subjective health on both immigrant and nonimmigrant participants.

### Support and later life well-being

Social support is known to play a critical preventive role in depression risk in later life. However, the effects of social support on mental health and depression vary depending on an individual’s social context and resources. Kahn and Antonucci [24] proposed an inclusive theoretical framework, social convoy theory, which highlights the role of social relationships in depression across the life span by emphasizing how social support changes over the life course and influences well-being. During stressful and transitional times, support needs change, and a person’s existing convoys may not adequately meet the demands of the situation. As a consequence, individuals may be at risk for loneliness, isolation, and depression [25]. According to this model, social relations are understood broadly and include multiple dimensions, such as social structure (size of the network, closeness of the person in the relationship), social satisfaction (family satisfaction and friendship satisfaction), and received and perceived social support. Some studies have emphasized the differing roles of perceived versus received support from friends and family in protecting against late-life depression [26].

The potential benefits of support are related to the personal meaning that individuals associate when perceiving support from others. *Perceived social support* refers to the *perception* that social relations are *accessible*,* effective*,* and sufficient* [8]. The belief that others are willing to help in times of need can reduce loneliness and isolation and increase overall well-being [27]. Longitudinal and cross-sectional studies indicate that perceived support increases feelings of safety, improves problem-solving skills, and reduces the negative effects of stress [1, 8].

In contrast to the subjective evaluation of perceived support, *received or enacted social support* refers to *actual supportive behaviors of others* [8]. Received social support can serve as a protective factor for overall well-being, and a lack of support can lead to powerlessness [27]. At the same time, receiving support may lead to feelings of helplessness and a lack of control over one’s life. In fact, cross-cultural studies, especially in the context of older adults refusing help and support in order not to be perceived as a burden, found that higher levels of enacted support, may be associated with higher depression rates [28]. For those already struggling with depression, the need for support can further increase feelings of vulnerability. Receiving support may challenge notions of independence and autonomy, leading to reduced well-being. These complex findings underscore the need for further studies exploring the influence of support for vulnerable older adults [29].

Relationship networks tend to include both family and friendships. Social convoy theory [24] suggests that with increasing age, support from family members may replace friendship support, particularly for individuals from collectivist cultures [30]. Studies exploring friendship satisfaction in Western cultures have revealed a positive relationship between friendship satisfaction and mental health, whereas in non-Western cultures, family satisfaction appears to play a more powerful role [31]. To investigate this complex relationship further, this study explores family and friendship satisfaction with support for a sample of older Turkish immigrants living in Germany with a comparative and matched sample of German nonimmigrants.

### Intersectional approach to gender and ethnicity

Immigration, age, gender, and ethnicity intersect with social relationships to shape vulnerability to depression [32]. These intersections play critical roles in shaping overall mental health and can contribute to an increased understanding of differences in later-life depression, particularly those related to ethnicity and immigration [33]. Intersectional theory emphasizes that social relations change according to factors such as gender and ethnicity. For example, older immigrants have been found to be more likely to trust and rely on family relationships than non-immigrants [34]. Similarly, Lui and Lin [35] have found that Black and Hispanic Americans report higher levels of depressive symptoms than their White counterparts in mid and late-life. However, to date we are not aware of a comprehensive study on the intersectionality of social relations related to gender and ethnicity in connection with depression among older adults.

### The current study

Given the global prevalence of depression and the debilitating consequences associated with later-life depression [36], this research explores the risk and protective factors among older Turkish immigrant and older nonimmigrant residents to clarify how ethnicity impacts depression later in life. More specifically, this study investigated the effects of social relationships, subjective health, age, gender, and ethnicity on depression rates among older Turkish immigrants in Germany and native Germans over the age of 75. The aims of the study were (a) to conduct a comparative analysis of depression rates among older Turkish immigrants living in Germany with a sociodemographic-matched group of older Germans; (b) to address the main effects of age, gender, ethnicity, subjective health, and social relationships on depression; and (c) to explore the ways in which ethnicity, gender, and social relationships intersect to shape vulnerability to depression.

## Method

### Participants

The participants in this study matched 195 older Turkish immigrants and 195 native Germans with similar demographic characteristics (please see Additional File Tables AF1-3 for matching procedures). Turkish participants had a mean age of 79.02 years (*SD* = 3.7), and German participants had a mean age of 79.01 years (*SD* = 3.5). See Additional File (Table AF3) for demographic information. Participants in the Turkish immigrant group were born in Turkey, spoke Turkish fluently, identified themselves as Turkish, and lived in Germany, while German participants were identified through the Nuremberg population register based on German citizenship. The inclusion criteria for both the Turkish immigrants and native German samples included (a) living in Nuremberg, Germany, and (b) being 75 years old or older. The exclusion criteria for both groups were (a) individuals with a neurodegenerative brain condition (i.e., dementia) and (b) those unable to respond to questions due to conditions such as aphasia or severe hearing impairment. A total of 390 women and men volunteered to participate in the study without compensation.

### Sampling strategy and matching procedure

The Turkish immigrant sample (*n* = 195) included volunteers who participated in face‒to‒face interviews conducted from August 2023 to April 2024. The initial sample included 202 participants, of whom *n* = 7 were excluded because of too many missing values. Convenience sampling procedures were employed to recruit participants via the time‒space method [14, 37]. Initially, locations frequented primarily by the older Turkish population were identified. Informational and invitation flyers were distributed at various Turkish organizations, such as Turkish Consulate, social and cultural organizations, local markets, restaurants, cafes, shops, family medicine offices, community events, breakfast gatherings, and mosques. The optimal times for recruiting individuals for the study were determined, Turkish communities were visited, and individuals were invited. The purpose of the study was explained to the participants, and informed consent was obtained. The interviews with those who agreed to participate in the study took approximately 30–60 min. German participants (*n* = 195) were drawn from a randomized sample of 10,000 addresses requested from the Nuremberg population register, and 2,393 people participated in this study online and via letters from July 2021 to December 2021. The sample included 2393 participants, of whom *n* = 100 were excluded because of missing gender information.

To test the role of social relationships and subjective health in depression among German and Turkish participants, twin datasets were created by matching the Turkish sample with demographically similar Native German participants. The matching criteria included the covariates of gender, age, relationship status, education, household income, perceived income, parental status and living alone [38, 39]. As part of this process, initially, raw data comparisons between Turkish immigrants and native Germans were conducted to identify group differences (please see Additional File Tables AF1). Subsequently, a propensity score was calculated (Additional File Tables AF2), and then manual matching was conducted (Additional File Tables AF3) to select the best matching strategy and reduce selection and information bias between the two groups. Both matching methods showed no significant differences between groups in terms of gender, age, relationship status, perceived income, parental status, and living alone status. However, significant differences remained between groups in terms of education and household income. For instance, the test of differences in household income between groups reduced notably after manual matching (*X²*_*raw data*_ = 297.1; *X²*_*propensity score matching*_ = 128.8; *X*^*2*^_*manual matching*_ = 64.1, *p* <.001; (please see Additional File Tables AF1-3), and this dataset was selected for further analysis. Given the complexity of the sociodemographic characteristics, achieving complete matching was challenging. The effects of sociodemographic characteristics on depression were explicitly controlled by including them as covariates in subsequent analyses.

### Measures

Surveys were administered in the participants’ native language. The original German version of the questionnaires was used for the German participants. For the Turkish version, we followed a translation and back-translation procedure to ensure both linguistic accuracy and conceptual equivalence. The initial translation from German to Turkish was conducted by bilingual researchers fluent in both languages, with bicultural background and advanced academic training in psychology and gerontology. To ensure accuracy, the translated questions were back-translated into German. The Turkish version was then pilot tested with a small group of native Turkish speakers to assess item clarity, validity, and cultural appropriateness. As no major issues were identified, the finalized Turkish version of questionnaires were then administered individually to the Turkish participants.

#### Social relations

Social relationships were evaluated via three measures: perceived social support, received social support, and social satisfaction. Higher scores on each dimension indicate greater levels of support and satisfaction.

*Received* social support was assessed with three items assessing enacted support from others: one item focused on received consolation *“Thinking about the last 12 months; how often did someone comfort you?”*, one item evaluated received advice *“How often did you get advice?”*, and *“How often did you get help?”* for receiving help [40]. The response options ranged from 1 (*“never”*) to 4 (*“often”*). High internal consistency was found in the Turkish sample (*α* = 0.96) and (*α* **=** 0.75) in the German sample.

*Perceived* social support was measured by F-SozU-14 items adapted from Fydrich et al. [41] (i.e., *“I can easily find someone to look after my apartment when I’m not there.” and “There are people who take me as I am without* restrictions”). The responses ranged from 1 (*“does not apply at all”*) to 5 (*“fully applies”*), and the internal consistency was satisfactory for both groups, with *α* = 0.89 (for Turkish participants) and *α* = 0.91 (for German participants).

*Social satisfaction* was evaluated by two items assessing satisfaction with family (i.e., *“How would you rate your relationship with your family overall at the moment?”*) and assessing friendship satisfaction (i.e., *“How would you rate your present relationship with your friends and acquaintances?”*). The participants responded on a five-point scale ranging from 1 (“*very bad*”) to 5 (“*very good*”). Then, they are recoded as 1 *(“low satisfaction”*), 2 (*“moderate satisfaction”*), and 3 *(“high satisfaction”*).

#### Depression

Depressive symptoms were evaluated with the 5-item short version of the Depression Inventory (BDI-V), adapted from applied version [42, 43]. This short form was derived from a simplified 20-item version of the original BDI, aiming to improve efficiency in large-scale or epidemiological studies. Schmitt et al. [44] reported that the simplified version demonstrated excellent internal consistency (Cronbach’s *α* = 0.94), which was even higher than that of the original BDI (*α* = 0.84). This supports the reliability of using a short form of the BDI-V for assessing depressive symptoms in non-clinical, cross-cultural research contexts. To ensure that the items clearly expressed culturally contoured meanings, item and scale analyses were performed for the 5 items and the total scale (see Additional File Table AF4). The respondents reported the questions (i.e., *“How often did you experience each mood or view over the past week?”*) on a 5-point scale ranging from 1 (*“never”*) to 5 (*“almost always”*). We intentionally chose not to dichotomize the data, as our research focused on the relationships between variables rather than on clinical diagnosis. Instead, depressive symptoms were treated as continuous variables, with an overall score calculated by summing the item responses, with higher scores indicating more severe depressive symptoms. The Cronbach’s alpha value was high (*α* = 0.89) for the Turkish sample and (*α* = 0.80) for the German sample.

#### Subjective health

Subjective health was evaluated by a single item (i.e., “*How would you describe your current general health as a percentage?”).* The participants responded on a point range from 0 to 100 (0 represents the worst health, and 100 indicates the best possible state of health). On the basis of previous studies, this parameter may be used both as a categorical variable [5] and a continuous numeric variable [45].

#### Control variables

Sociodemographic variables, including education, household income, perceived income, marital and parental status, religion, attending religious events, and living status, were used as control variables.

### Statistical procedure

Chi-square and t tests were performed to examine potential differences due to gender and ethnicity on demographic data and means, respectively (see Table 1). A bivariate correlation analysis was conducted to explore the relationships among variables related to depression by gender, and ethnicity (see Additional File Tables AF5-6). A hierarchical regression approach was subsequently conducted, with predictors introduced sequentially across different models (see Table 2). Model 1 was composed of the variables gender, age, ethnicity, and subjective health to address the main effects of depression. Received and perceived social support, satisfaction with family and friendship are included in Model 2, followed by interaction effects between gender, ethnicity, age, and other predictors in Model 3. Covariates such as education, household income, and relationship status were added to Model 4. To reduce issues related to multicollinearity, centered scores were utilized. Moreover, each interaction effect was rigorously assessed independently. The results were deemed statistically significant when the *p* value was less than 0.05 (*p* <.05).

This study is exploratory, the lack of previous studies that would allow estimating sample sizes for comparisons means that no a priori power analysis was conducted. However, in accordance with recommended standards for comparative and correlational studies in the social and health sciences, approximately 200 participants were included for each subsample [46]. Post-hoc power analyses, that is, power calculations performed after data collection and hypothesis testing, were not conducted because they have limited validity in the literature and do not provide additional information [47]. Therefore, Cohen’s d and R² values ​​were calculated and reported as an alternative method to address the robustness and magnitude of the observed effects [48].

## Results

### Descriptive and bivariate analysis

Table AF3 shows the descriptive statistics of the sociodemographic variables by gender and ethnicity. Although the present study identified two subsamples that are comparable and similar in many key sociodemographic domains, it demonstrates that perfect matching between Turkish immigrants and native Germans in terms of education and income levels is not feasible due to social and historical inequalities. Germans had a higher education (*X*^*2*^ = 10.8, *p* =.013) and income level (*X*^*2*^ = 64.1, *p* <.001) than Turkish participants in this study. Turkish immigrants scored higher on depression than German participants (*t* = 7.23, *p* <.001) and reported lower perceived social support (*t* = −3.34, *p* <.001), lower satisfaction with family relations (*t* = −6.03, *p* <.001), and lower subjective health (*t* = −3.30, *p* <.001). Compared with men, women also scored higher on depression (*t* = 2.18, *p* =.030); had higher scores on received social support (*t* = 3.48, *p* <.001) and satisfaction with friendships (*t* = 2.84, *p* =.005) in both cultures (Table 1). 

**Table 1:** Mean scores of measures by ethnicity and gender

	Turkish Sample (*n* = 195)		German Sample (*n* = 195, matched)		Total Sample (*N* = 390)		Test of differences between Female and Male	Total Sample (*N* = 390)		Test of differences between Turkish and German	Cohen`s d Effect Size
Main predictors of depression	Female (*n* = 105)	Male (*n* = 90)	Female (*n* = 105)	Male (*n* = 90)	Female (*n* = 210)	Male (*n* = 180)		Turkish (*n* = 195)	German (*n* = 195)		
	Mean (SD)	Mean (SD)	Mean (SD)	Mean (SD)	Mean (SD)	Mean (SD)	Δ Subsamples	Mean (SD)	Mean (SD)	Δ Subsamples	
Beck Depression Inventory^a^	2.90 (1.1)	2.68 (1.2)	2.21 (0.86)	1.97 (0.63)	2.55 (1.0)	2.33 (1.0)	t(388) = 2.18*	2.80 (1.1)	2.1 (0.76)	t(388) = 7.23***	d_1_ = 0.70; d_2_ = 0.74, d_3_ = 0.22; d_4_ = 0.74
Enacted Received Support^b^	2.66 (1.2)	2.26 (1.1)	2.69 (0.76)	2.41 (0.73)	2.67 (0.98)	2.34 (0.93)	t(388) = 3.48***	2.47 (1.1)	2.6 (0.76)	t(388) = − 0.88	d_1_ = 0.03; d_2_ = 0.16; d_3_ = 0.34; d_4_ = 0.14
Perceived Social Support^a^	3.84 (0.68)	3.81 (0.65)	4.11 (0.72)	4.02 (0.82)	3.97 (0.71)	3.92 (0.75)	t(388) = 0.701	3.82 (0.66)	4.07 (0.77)	t(388)= −3.34***	d_1_ = 0.39; d_2_ = 0.28; d_3_ = 0.07; d_4_ = 0.35
Family Satisfaction^c^	2.03 (0.79)	1.97 (0.73)	2.43 (0.66)	2.43 (0.67)	2.23 (0.76)	2.20 (0.73)	t(388) = 0.439	2.00 (0.76)	2.44 (0.67)	t(388)= −6.03***	d_1_ = 0.55; d_2_ = 0.66; d_3_ = 0.04; d_4_ = 0.61
Friend Satisfaction^c^	2.25 (0.74)	1.94 (0.72)	2.07 (0.59)	1.98 (0.64)	2.16 (0.68)	1.96 (0.68)	t(388) = 2.84**	2.11 (0.75)	2.03 (0.61)	t(388) = 1.18	d_1_ = 0.27; d_2_ = 0.06; d_3_ = 0.29; d_4_ = 0.12
Subjective Health^d^	61.68 (26.8)	65.57 (23.2)	68.67 (19.3)	73.37 (16.8)	65.17 (23.5)	69.47 (20.5)	t(388) = − 1.90	63.47 (25.2)	70.84 (18.3)	t(388) =−3.30***	d_1_ = 0.30; d_2_ = 0.38; d_3_ = 0.19; d_4_ = 0.33

Descriptive statistics are shown as the means with standard deviation in parentheses

*p <.05

**p <.01

***p <.001

^a^Scale range 1–5

^b^Scale range 1–4

^c^Scale range 1–3

^d^ Scale range 0−100

d_1_ = Turkish Female - German Female

d_2_ = Turkish Male - German Male

d_3_ = Female - Male

d_4_ = Turkish sample - German sample

The bivariate correlations of the study variables by gender for both samples are shown in Additional File Tables AF5-6. Increasing age was also significantly associated with depression for German women only. Higher levels of received social support were associated with higher depression scores for Turkish participants and German women, whereas there was no association for German men. Perceived social support, and social satisfaction were negatively correlated with depression in the Turkish sample and German men but not in the German women. Furthermore, a negative correlation between subjective health and depression was found for the Turkish sample and the German women, but there was no significant correlation for the German men.

### Hierarchical regression analysis

Hierarchical regression analysis was conducted to examine the associations with the predictors of depression (Table 2). Model 1 explored the effects of the individual-level variables, age, gender, ethnicity differences and also subjective health. The results of the analysis accounted for 27.7% (*F* = 38.3, *p* <.001) of the variance, which was statistically significant. A lower level of subjective health combined with the experience of immigration appears to be associated with a greater risk late life for depression (*β*_*ethnicity: Turkish*_ = − 0.280, *p* <.001; *β*_*subjective health*_ = − 0.388, *p* <.001).

Model 2 estimated the regression coefficients of the three components of social relationships at 40.0% (*F* = 33.4, *F*_*Change*_ = 20.6, *p* <.001, *p*_*F change*_ < 0.001), a statistically significant finding. Received enacted support is associated with greater risk of depressive feelings (*β*_*received support*_ = 0.246, *p* <.001), whereas greater perceived support, satisfaction with family, and friendship relationships appear to protect against depression (*β*_*perceived social support*_ = − 0.180, *p* <.001; *β*_*family satisfaction*_ = − 0.128, *p* =.005; *β*_*friendship satisfaction*_ = − 0.118, *p* =.011). 

**Table 2:** Regression coefficients on depression

Variables	Model 1			Model 2			Model 3		
	B	SE	β	B	SE	β	B	SE	β
(Constant)	1.518	0.151		1.191	0.144		1.160	0.148	
Gender (R = woman)	− 0.161	0.089	− 0.079	− 0.138	0.083	− 0.067	− 0.120	0.115	− 0.059
Ethnicity (R = Turkish)	− 0.572	0.089	− 0.280***	− 0.504	0.087	− 0.247***	− 0.542	0.114	− 0.265***
Age (*R* = 75–79)	0.121	0.096	0.055	0.039	0.089	0.018	0.043	0.087	0.020
Subjective Health	− 0.018	0.002	− 0.388***	− 0.013	0.002	− 0.291***	− 0.013	0.002	− 0.291***
Enacted Received Support (ERS)				0.260	0.044	0.246***	0.192	0.066	0.182**
Perceived Support (PS)				− 0.254	0.065	− 0.180***	− 0.265	0.065	− 0.189***
Family Satisfaction (FS)				− 0.176	0.063	− 0.128**	− 0.371	0.078	− 0.271***
Friendship Satisfaction (FrS)				− 0.176	0.069	− 0.118*	− 0.159	0.068	− 0.106*
Ethnicity *FS							0.446	0.113	0.210***
Ethnicity *ERS							0.174	0.120	0.091
Gender* Ethnicity							− 0.033	0.160	− 0.014
Gender*ERS							0.197	0.101	0.122
Gender* Ethnicity*ERS							− 0.612	0.182	− 0.210***
Adj. R^2^	0.28			0.40			0.43		
ΔR^2^				0.13			0.04		
F Change				20.6			5.1		

Coefficients printed in bold are significant (* *p* <.05; ** *p* <.01; *** *p* <.001); *N* = 390

In addition, we tested all effects of the control variables (relationship status, education, income, and perceived income, parental status, religion, the attending of the religion events, living alone). No significant associations were found with higher levels of depression, however, only interaction effect of gender and received support became significant on depression (*p* =.040, β = 0.130). Also there were no interaction effects of subjective health by gender, age or culture on depression. (*Adjusted R²* = 0.43, *ΔR*^*2*^ = 0.01, *F Change* = 1.1, *p*_*F change*_ = 0.39)

*Cl* confidence interval, *SE* standard error, *β* standardized β coefficients, *Adj. R*^*2*^ Adjusted R square, *ΔR*^*2*^ Adjusted R² Change

**Fig. 1:**
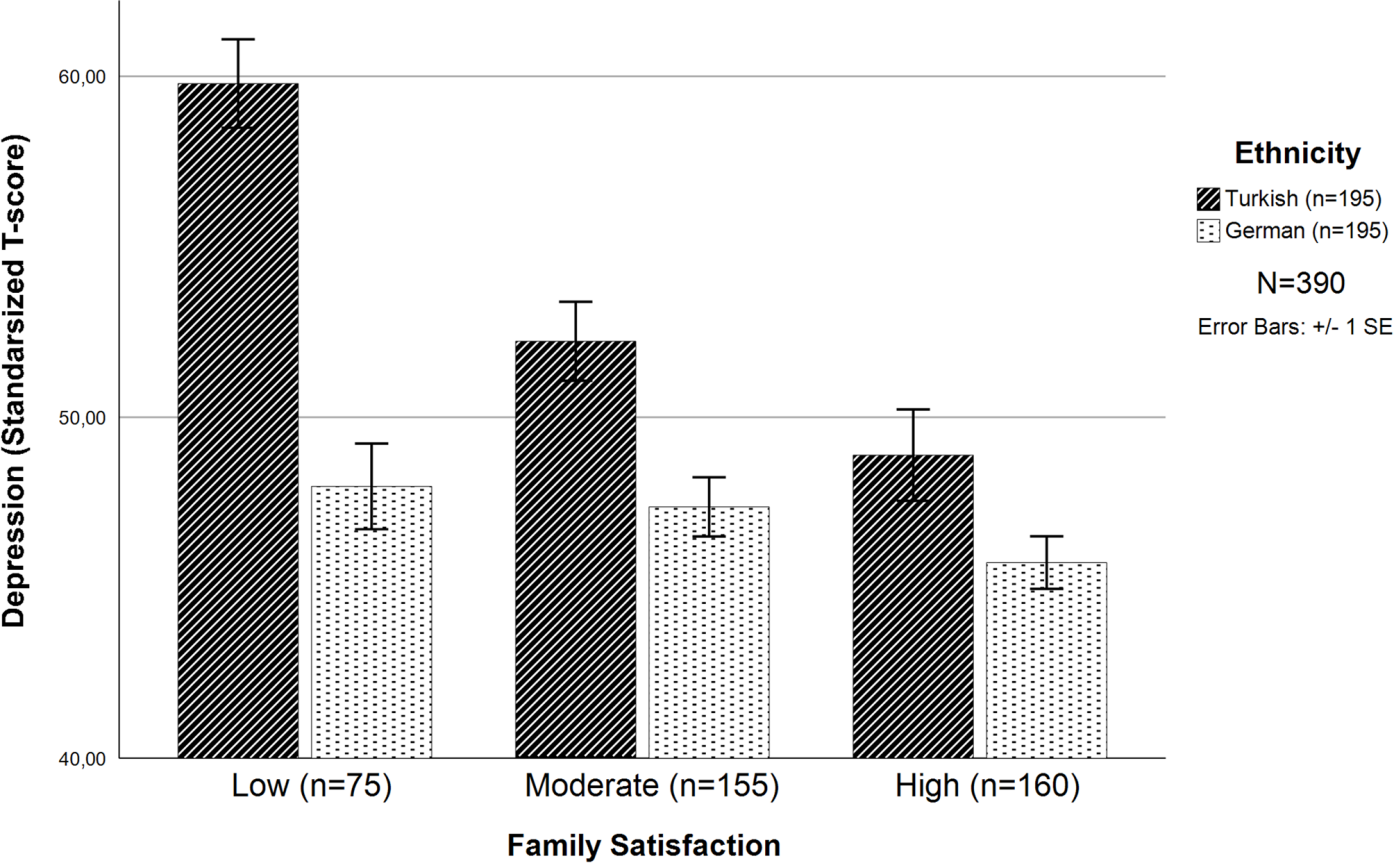
Two-way interaction effect of ethnicity and family satisfaction on depression (*T-score*, *SE*, *N* = 390). (*p* <.001, *β* = 0.200). *Note*: T-scores represent standardized values from the depression scale (*M* = 50, *SD* = 10), not statistical t-values

**Fig. 2:**
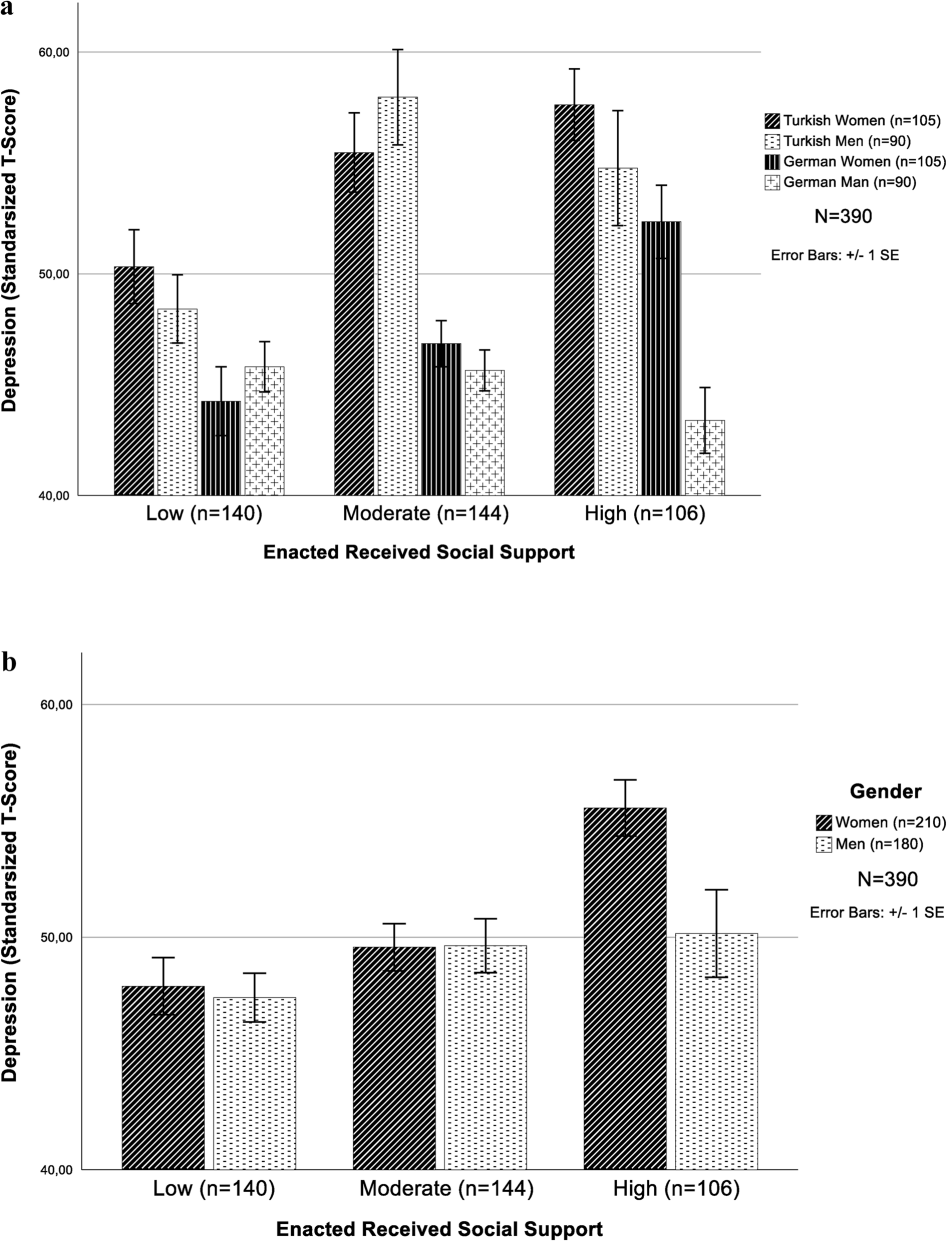
**a** Three-way interaction effect of enacted received support, gender, and ethnicity on depression (*T-score*, *SE*; *N* = 390). (*p* < .001, *β* = -.214). **b** Two-way interaction effect of gender and enacted received support on depression (*T-score*, *SE*; *N* = 390). (*p* = .040, *β* =.130). *Note:* T-scores represent standardized values from the depression scale (*M* = 50, *SD* = 10), not statistical t-values

The interaction effect of all variables on depression by gender, age and ethnicity (Model 3) explained 43.0% (*F* = 23.6, *F*_*Change*_ = 5.1, *p* <.001, *p*_*F change*_ < 0.001) of the variance and was statistically significant. The positive association between satisfaction with family relationships and depression was stronger for Turkish participants than for German participants (*β*_*ethnicityXfamily satisfaction*_ = 0.210, *p* <.001; Fig. 1). Low satisfaction with family relations is a risk factor in the Turkish sample but not in the German sample. The three-way interaction effect of gender, ethnicity, and received support on depression was found to be significant (*β*_*genderXethnicityXreceived support*_ = − 0.210, *p* <.001; Fig. 2a), although no significance was found for the two-way interaction effect per (*β*_*genderXethnicity*_ = − 0.014, *β*_*genderXreceived support*_ = 0.122, *β*_*ethnicityXreceived support*_ = 0.091; *p* >.005) in Model 3.

Women’s depression scores indicate a similar trend for both samples, even if their depression levels vary, with those who have higher depression rates also reporting higher levels of received support. However, the depression scores for Turkish men indicate differences when receiving more support, whereas there is almost no such difference among German men (Fig. 2a). The effects of the control variables (i.e., education, income, and perceived income, parental status, relationship status, living alone, religion, attending religion events) were tested in Model 4. No significant associations were found with higher levels of depression, but the two-way interaction effect of received support by gender became significant (*β*_*genderXenacted received support*_ = 0.130, *p* =.040; Fig. 2b). Higher levels of received support are a greater risk factor in women but not in men. The results of Model 4 explained 43.1% of the variance (*F* = 15.04, *F*_*Change*_ = 1.1, *p* <.001, *p*_*F Change*_ = 0.386).

## Discussion

The primary goal of this study was to compare depression among older Turkish immigrants living in Germany and sociodemographic-matched older German natives. More specifically, this study focused on gender, age, subjective health, and ethnicity, exploring the main and moderation effects of social relations. Consistent with other research suggesting that immigrants are at risk for depression, Turkish immigrants in this study scored higher than their non-immigrant counterparts on depression. Women also reported higher depression scores than men. High levels of satisfaction with friendships and subjective health were found to play a protective role against depressive feelings in both groups. Moderating factors included family satisfaction and receiving social support, with low satisfaction with family relations associated with a higher risk of depression in the Turkish sample but not in the German sample. Higher satisfaction with family relationships thus played a protective role for Turkish participants but not for German participants. In contrast, receiving greater support was associated with a greater risk of depressive symptoms, both in Turkish and German women, but to a lesser degree in Turkish men and not at all in German men. This study contributes to an improved understanding of the moderation effect of receiving social support by gender and ethnicity and the moderation effect of family satisfaction by ethnicity on depression in later life.

### The impact of gender and ethnicity on depression

The findings shed new light on the role of gender, age, and ethnicity as risk factors for late-life depression. Consistent with related studies, older immigrants were found to be at greater risk for depression than nonimmigrants of similar age and social demographic characteristics, such as income, education and/or family status. Several intersecting factors shape vulnerability, including immigration, linguistic isolation, social, emotional, and financial resources, as well as health and well-being [2, 49]. The immigrant participants in this study had continued to live in Germany postretirement (at age 65, i.e., 10 years or older). However, most of the participants lived in diasporic communities and had limited contact with the host culture. Socioeconomic status also plays a role in shaping well-being. In this study, despite sample matching, the education and income levels of German native participants were higher than those of Turkish participants, a factor that may have contributed to depression risk. Immigration can lead to a lifetime of cumulative disadvantage, which may include poverty, a lack of access to culturally sensitive health care, language barriers, and additional immigration-related stressors. The intersection of these factors can significantly increase the risk for late-life depression and other mental health concerns [6, 50].

### Subjective health and depression

Numerous studies have shown that subjective health plays a powerful role in depression risk [5]. Subjective health assessment is one of the measures, as they tend to encompass physical health, mental health, as well as life circumstances. In this study, subjective health was also found to be one of the main predictors of depression with women scoring lower, and Turkish participants reporting significantly lower overall subjective health than native German participants. These results highlight the importance of addressing the subjective vulnerability of aging immigrants, particularly older immigrant women [22].

### The role of social relations in depression

Social convoy theory can be useful in understanding the dynamic nature of social relationships and depression, particularly in later life. According to this model, elements of social support, including social networks, perceived and received social support, and social support satisfaction [24], intersect to shape well-being. Perceived support was identified as the strongest predictor of depression, particularly in later life, in this study. In this study, older native Germans reported greater perceived support than their Turkish immigrant counterparts, who also scored higher on depression measures. Perceived support focuses on the belief that one’s social connections are accessible, helpful, and satisfactory [8]. Trusting that others provide help and support can reduce loneliness and increase feelings of safety and security, leading to more robust mental health. Higher levels of perceived social support can also mitigate depression and bolster coping and self-regulatory mechanisms, particularly in the face of late-life health challenges [51].

Research has focused predominantly on the impact of perceived support when examining the association between social relationships and depression [29]. There are, however, still gaps regarding the effects of other dimensions of support such as enacted received support. Despite the protective effects of perceived support, some studies have emphasized that the provision of enacted support may, in fact, lead to sensitization and to an increased depressive response [28]. Personal and cultural factors intersect to shape support expectations as well as satisfaction with both perceived and received support [52]. In this study, enacted received support was positively related to higher rates of depression. For older adults, being confronted with the need to receive help and support from others may raise awareness and concerns about one’s personal lack of control, dependency and helplessness [28].

### Intersection of gender and ethnicity with received social support

Gendered socialization shapes social relationships. For example, numerous studies have shown that women tend to have wider support networks and a greater number of social connections [7, 50]. In this study, women received more support than men, but also had higher rates of depression. Gender-specific social role expectations and cultural values and norms may contribute to this finding. For example, related studies have found that higher levels of received support can result in feelings of dependance and indebtedness, which, in turn, could negatively influence overall well-being [53].

Studies have also revealed that culturally inappropriate or nonempathetic provision of enacted support may have a negative impact [29]. For example, in this study, scores on received support were associated with higher depression scores for both German and Turkish female participants. For men, the findings indicated a more complex relationship. For Turkish men, as support increased, depression rates also increased; however, in German men with high levels of support, depression rates decreased. For German men, low and moderate levels of support were not associated with depression rates; high levels of support, on the other hand, were related to lower rates of depression. Clearly, gendered expectations intersect with cultural socialization to influence the relationship between support and mental health.

Overall, women tend to experience greater health challenges than men, particularly in later life. Ang and Malhotra [29] proposed that one reason women experience greater vulnerability to depression is related to the social support they receive. This heightened sensitivity could be attributed to gender-specific mechanisms and expectations of support. One finding of this study relates to the negative relationship between receiving support and depression in women late in life. To our knowledge, this study is the first to address the three-way interaction effects of gender, ethnicity, and receiving social support on mental health among older Turkish immigrants compared with older nonimmigrants. This finding warrants further investigation. Additionally, in this study, when social support was incorporated at the item level rather than as a composite score, receiving support in the form of “*received consolation*” was associated with a greater likelihood of depression.

### Intersection of ethnicity with family satisfaction

Across the life course, people have a need for affiliation and a sense of belonging. This need tends to be satisfied through relationships with family and friends. Satisfaction with social relationships, family or friendships may contribute to maintaining well-being. In this study, both higher family satisfaction and friendship satisfaction play a protective role against depression. Additionally, gender comparison data indicated that women scored higher than men on satisfaction with friendships, however, cultural variations were not found on this measure. No gender differences were found in satisfaction with family relationships; however, ethnic differences were identified. Satisfaction with family relationships was significantly greater for German participants, and low levels of family satisfaction were, in fact, identified as risk factors for depression in Turkish participants. As satisfaction with family relationships increased for the Turkish sample, depression rates decreased, although overall, Turkish immigrants still scored higher on depression. In other words, family satisfaction did not appear to be a sufficient protective factor against depression. It appears that family satisfaction plays a key role in preventing depression; however, cultural expectations must be taken into consideration.

Ethnic values and norms shape expectations regarding family relationships and support. Turkish family structure tends to more collectivist and group focused with an emphasis on filial piety [49]. Studies examining the mediation and moderation effects of family relationships on depression have been conducted primarily in Asian cultures [13, 50]. In general, the findings from these studies indicate that older immigrants are reliant on help from family to manage later life challenges. If sufficient help exists and is provided in a culturally appropriate manner, it can help older immigrants maintain their well-being [13]. This study contributes to literature by examining the interaction effect family satisfaction and depression by conducting a comparative analysis of native Germans and older Turkish immigrants.

### Limitations

Although this study contributes to an improved understanding of the concomitant depressive symptoms of older Turkish immigrants and nonimmigrants living in Germany, a few caveats should be considered when the findings are interpreted. First, the cross-sectional nature of the study prevents any conclusions regarding the temporal sequence of the observed effects. Longitudinal data will be needed to improve the understanding of the underlying moderation and mediation processes. Second, this study is unique in that we included immigrant and nonimmigrant adults over the age of 75. While the nonimmigrants could be matched from a large and heterogeneous sample that is based on local registration offices, the immigrants who participated in the study reflected a convenience sampling procedure via the time‒space method and consisted exclusively of individuals of Turkish origin. In this vein, the present findings are specific to older Turkish immigrants and should be interpreted within this cultural and demographic context. Thus, the size, sociodemographic characteristics, and age range of the participants may limit the generalizability of the findings, particularly with respect to the prevalence of depressive symptoms in this study. Third, we collected data for the two samples in this study at different times. This raises the possibility that changes over time could have influenced the differences observed between the groups. However, our analysis focused on the relationships between structural and contextual factors and depression, which are rooted in long-standing socioeconomic inequalities. Therefore, we believe that temporal effects do not significantly influence the main conclusions of our findings. Nevertheless, future studies may take this issue into account when interpreting or designing comparative analyses. Finally, the age at immigration, language proficiency, or reason for immigration was not examined. Future research should focus on detailed immigration histories for more nuanced analyses.

## Conclusion

This research explored the mental health concerns of older Turkish immigrants in comparison to a matched sample of native German older adults. Given the increase in the older adult population, the exploration of risk and protective factors for late-life depression is crucial. Ethnicity and gender play powerful roles in mental health. In this study, Turkish participants were found to be more depressed than native German participants. Factors such as low education, income, and late-life loss, however, cannot be discounted. There is a need to further understand the ways in which the mental health of older immigrants is shaped, in part, by satisfaction with social relationships and the complexity of beliefs about perceived and received support.

Social support expectations are culturally nuanced and gendered and have implications for the development of culturally sensitive mental health services. Barriers in providing effective services for older adults, particularly older immigrant adults, are well documented [1, 15, 54]. The results of this study can be helpful in the development of culturally competent services and programs. The findings increase awareness of culturally shaped expectations regarding family and social support in later life. In multicultural urban settings, services such as bilingual counseling, culturally matched peer support groups, and preventive outreach efforts may help strengthen perceived social support and reduce vulnerability to depression among older immigrant populations. Further studies exploring the well-being of immigrants in late life should consider immigration stress as well as the bidirectional effects of self-regulation strategies during the acculturation process on their psychological health in later life.

## Supplementary Information

Supplementary Material 1.

## Data Availability

The data supporting the findings of this study are available from the corresponding author upon reasonable request.
