# Supplementary material for: Depression in older Turkish immigrants and natives in Germany: a comparative analysis of risk and protective factors
Source: BMC Public Health. 2025 Oct 31;25:3707. doi: 10.1186/s12889-025-24954-9 (PMC12577309; doi:10.1186/s12889-025-24954-9)
Supplement: Supplementary file 1 — Supplementary Material 1. [file 12889_2025_24954_MOESM1_ESM.docx]

**Additional file**

**DEPRESSION IN OLDER TURKISH IMMIGRANTS AND NATIVES IN GERMANY: A COMPARATIVE ANALYSIS OF RISK AND PROTECTIVE FACTORS**

N. Tugba Bahar^a,b*^, Jasmin Tahmaseb McConatha^c^, Frieder R. Lang^a^

^a^Institute of Psychogerontology, Universität Erlangen-Nürnberg, Nuremberg, Germany

^b^Department of Health Care Services, Vocational High School of Health Services, Giresun University, Giresun, Turkey

^c^Department of Psychology, West Chester University of Pennsylvania, West Chester, PA 19383, USA

This file contains supplementary information material including additional analyses conducted in order to support findings reported in the manuscript entitled “Depression in older Turkish immigrants and natives in Germany: a comparative analysis of risk and protective factors” Additional file Table AF1-3 explain a data matching strategy and procedures. Additional file Table AF4 displays the item-total correlations analysis of K5-Beck Depression Inventory. Additional file Table AF5-6 show a summary of correlation results of Turkish and German sample by gender.

**Data Matching Strategy and Procedure**

**Additional file Table AF1-3**

Initially, when the research sample was divided by gender and ethnicity, there was a significant difference in sample size between the Turkish immigrant (n=195) and German native (n=2393) older adult groups. Initially, missing data were checked, and due to missing gender information for 100 participants in the German sample, we removed these 100 participants from the sample. Then, we compared between groups for sociodemographic characteristics on the existing data set of Turkish immigrants and Germans (please see Supplemental Table 1). Nevertheless, to decrease potential selection bias between groups and to make more robust causal inferences, we used matching strategies to find the closest possible sub-samples.

We used propensity score matching (PSM) and manual matching to compare each immigrant to their German counterparts with the similar characteristics. For matching criterias, we determined the covariates as gender, age, relationship status, the levels of education, household net income, perceived income, parental status and living alone (Deng & Law, 2020; Rupprect et al., 2022).

Propensity Score Matching (PSM) is a widely used method to decrease bias between groups in studies (Arsenijevic & Groot, 2018). Using the PSM method, each participant's propensity score was calculated and participants from both groups were matched based on these scores. 1:1 nearest neighbor matching method was used as the most appropriate participants for matching the data. After PSM, differences in sociodemographic variables between immigrants and native groups were control by using chi-squared test (Yun et al., 2022) (please Supplemental Table 2). Alternative matching approaches, in addition to PSM, are suggested when the results are sensitive and the researcher has reservations about their validity. This benefits for the creation of more sensitive and compatible couples in the matching process (Caliendo & Kopeinig, 2008). In this vein, the manual-matching technique was used in order to find the best selection strategy.

In the manual matching, we conducted the command *`Select Cases; if condition is satisfied`* on SPSS for each Turkish people and their German counterparts. After manual matching, chi-squared test for categorical variables were performed again (Please see Supplemental Table 3). In the data set obtained by manual matching, the differences between socio-demographic characteristics were well balanced, and intergroup bias was reduced more effectively for all variables (please see Supplemental Table 2 and 3). This situation can be explained by the fact that the researcher can evaluate the characteristics of individuals in both groups in more detail and carefully during manual matching. Since the PSM method is based on automatically calculated propensity scores, in some cases small differences between these scores may be likely to lead to matching errors. To sum up, we decided to use obtained by manual matching in this study.

**Additional references**

Arsenijevic, J., & Groot, W. (2018). Lifestyle differences between older migrants and non-migrants in 14 European countries using propensity score matching method. *International journal of public health*, *63*, 337-347.

Caliendo, M., & Kopeinig, S. (2008). Some practical guidance for the implementation of propensity score matching. *Journal of economic surveys*, *22*(1), 31-72.

Deng, Z., & Law, Y. W. (2020). Rural-to-urban migration, discrimination experience, and health in China: Evidence from propensity score analysis. *PLoS One*, *15*(12), e0244441.

Rupprecht, F. S., Sabatini, S., Diehl, M., Gerstorf, D., Kaspar, R., Schilling, O. K., & Wahl, H. W. (2022). Awareness of age-related change in the context of major life events. *Frontiers in Psychiatry*, *13*, 954048.

Yun, J., Lee, Y., & Lee, H. J. (2022). A comparison of health-related quality of life and personal, social, and environmental factors of older adults according to a residential area: A propensity score matching analysis. *Quality of Life Research*, *31*(9), 2631-2643.

**Table AF1.** Sample Description by gender and ethnicity **(Before Matching)**

|  | **Turkish Sample**  **(N=195)** | | **German Sample**  **(N=2293)** | | **Total Sample**  **(N=2488)** | | **Test of differences between Turkish and German** |
| --- | --- | --- | --- | --- | --- | --- | --- |
| Measure | **Female**  **(N=105)** | **Male**  **(N=90)** | **Female**  **(N=1256)** | **Male**  **(N=1037)** | **Female (N=1361)** | **Male (N=1127)** |  |
|  | N (%) | N (%) | N (%) | N (%) | N (%) | N (%) | Δ Subsamples |
| Age Group |  |  |  |  |  |  | X^2^ = 87.2*** |
| 75-79 | 77 (73.3) | 57 (63.3) | 453 (36.1) | 354 (34.1) | 530 (38.9) | 411 (36.5) |  |
| 80+ | 28 (26.7) | 33 (36.7) | 801 (63.8) | 682 (65.8) | 829 (60.9) | 715 (63.4) |  |
| Missing Answer |  |  | 2 (.2) | 1 (.1) | 2 (.1) | 1 (.1) |  |
| Relationship Status |  |  |  |  |  |  | X^2^ = 7.0** |
| Have not partner | 53 (50.5) | 14 (15.6) | 758 (60.4) | 238 (23.0) | 811 (59.6) | 252 (22.4) |  |
| Have a partner | 52 (49.5) | 76 (84.4) | 480 (38.2) | 782 (75.4) | 532 (39.1) | 858 (76.1) |  |
| Missing Answer |  |  | 18 (1.4) | 17 (1.6) | 18 (1.3) | 17 (1.5) |  |
| Level of Education |  |  |  |  |  |  | X^2^ = 82.4*** |
| Unable to read and write | 20 (19.0) | 1 (1.1) | 28 (2.2) | 16 (1.5) | 48 (3.5) | 17 (1.5) |  |
| Elementary school | 64 (61.0) | 56 (62.2) | 584 (46.5) | 426 (41.1) | 648 (47.6) | 482 (42.8) |  |
| Secondary school | 11 (10.5) | 12 (13.3) | 432 (34.4) | 249 (24.0) | 443 (32.5) | 261 (23.2) |  |
| (technical) high school and over | 10 (9.5) | 21 (23.3) | 165 (13.1) | 277 (26.7) | 175 (12.9) | 298 (26.4) |  |
| Missing Answer |  |  | 47 (3.7) | 69 (6.7) | 47 (3.5) | 69 (6.1) |  |
| Household net income |  |  |  |  |  |  | X^2^ = 297.1*** |
| Less than € 1000 | 31 (29.5) | 14 (15.6) | 95 (7.6) | 17 (1.6) | 126 (9.3) | 31(2.8) |  |
| €1001 - €1500 | 45 (42.9) | 38 (42.2) | 181 (14.4) | 71 (6.8) | 226 (16.6) | 109 (9.7) |  |
| €1501 - €2000 | 23 (21.9) | 30 (33.3) | 245 (19.5) | 109 (10.5) | 268 (19.7) | 139 (12.3) |  |
| More than €2001 | 6 (5.7) | 8 (8.9) | 592 (47.1) | 748 (72.1) | 598 (43.9) | 756 (67.1) |  |
| Missing Answer |  |  | 143 (11.4) | 92 (8.9) | 143 (10.5) | 92 (8.2) |  |
| Perceived Income |  |  |  |  |  |  | X^2^ = 90.5*** |
| My income is less than my expenses | 22 (21.0) | 6 (6.7) | 98 (7.8) | 49 (4.7) | 120 (8.8) | 55 (4.9) |  |
| My income equals my expenses | 32 (30.5) | 22 (24.4) | 114 (9.1) | 89 (8.6) | 146 (10.7) | 111 (9.8) |  |
| My income more than my expenses | 51 (48.6) | 62 (68.9) | 1029 (81.9) | 883 (85.1) | 1080 (79.4) | 945 (83.9) |  |
| Missing Answer |  |  | 15 (1.2) | 16 (1.5) | 15 (1.1) | 16 (1.4) |  |
| Parental Status |  |  |  |  |  |  | X^2^ = 25.7*** |
| No | 8 (7.6) | 2 (2.2) | 214 (17.0) | 170 (16.4) | 222 (16.3) | 172 (15.3) |  |
| Yes | 97 (92.4) | 88 (97.8) | 1039 (82.7) | 864 (83.3) | 1136 (83.5) | 952 (84.5) |  |
| Missing Answer |  |  | 3 (.3) | 3 (.3) | 3 (.2) | 3 (.3) |  |
| Living Alone |  |  |  |  |  |  | X^2^ = 20.2*** |
| No | 65 (61.9) | 80 (88.9) | 515 (41.0) | 784 (75.6) | 580 (42.6) | 864 (76.7) |  |
| Yes | 40 (38.1) | 10 (11.1) | 708 (56.4) | 237 (22.9) | 748 (55.0) | 247 (21.9) |  |
| Missing Answer |  |  | 33 (2.6) | 16 (1.5) | 33 (2.4) | 16 (1.4) |  |
| Descriptive statistics are shown as the number together with % in parentheses. *p< .05; **p< .01; ***p< .001 | | | | | | | |

**Table AF2.** Sample Description by gender and ethnicity **(After PSM)**

|  | **Turkish Sample**  **(N=195)** | | **German Sample**  **(N=195)** | | **Total Sample**  **(N=390)** | | **Test of differences between Turkish and German** |
| --- | --- | --- | --- | --- | --- | --- | --- |
| Measure | **Female**  **(N=105)** | **Male**  **(N=90)** | **Female**  **(N=105)** | **Male**  **(N=90)** | **Female (N=210)** | **Male (N=180)** |  |
|  | N (%) | N (%) | N (%) | N (%) | N (%) | N (%) | Δ Subsamples |
| Age Group |  |  |  |  |  |  | X^2^ = .00  ΔX^2^ =87.2 |
| 75-79 | 77 (73.3) | 57 (63.3) | 77 (73.3) | 57 (63.3) | 154 (73.3) | 114 (63.3) |  |
| 80+ | 28 (26.7) | 33 (36.7) | 28 (26.7) | 33 (36.7) | 56 (26.7) | 66 (36.7) |  |
| Relationship Status |  |  |  |  |  |  | X^2^ = .42  ΔX^2^ =6.54 |
| Have not partner | 53 (50.5) | 14 (15.6) | 52 (49.5) | 9 (10.0) | 105 (50.0) | 23 (12.8) |  |
| Have a partner | 52 (49.5) | 76 (84.4) | 53 (50.5) | 81 (90.0) | 105 (50.0) | 157 (87.2) |  |
| Level of Education |  |  |  |  |  |  | X^2^ = 41.8***  ΔX^2^ =40.6 |
| Unable to read and write | 20 (19.0) | 1 (1.1) | 2 (1.9) | 2 (2.2) | 22 (10.5) | 3 (1.7) |  |
| Elementary school | 64 (61.0) | 56 (62.2) | 46 (43.8) | 34 (37.8) | 110 (52.4) | 90 (50.0) |  |
| Secondary school | 11 (10.5) | 12 (13.3) | 36 (34.3) | 27 (30.0) | 47 (22.4) | 39 (21.7) |  |
| (technical) high school and over | 10 (9.5) | 21 (23.3) | 21 (20.0) | 27 (30.0) | 31 (14.8) | 48 (26.7) |  |
| Household net income |  |  |  |  |  |  | X^2^ = 128.8***  ΔX^2^ =168,3 |
| Less than € 1000 | 31 (29.5) | 14 (15.6) | 8 (7.6) | 3 (3.3) | 39 (18.6) | 17 (9.4) |  |
| €1001 - €1500 | 45 (42.9) | 38 (42.2) | 23 (21.9) | 10 (11.1) | 68 (32.4) | 48 (26.7) |  |
| €1501 - €2000 | 23 (21.9) | 30 (33.3) | 21 (20.0) | 12 (13.3) | 44 (21.0) | 42 (23.3) |  |
| More than €2001 | 6 (5.7) | 8 (8.9) | 53 (50.5) | 65 (72.2) | 59 (28.1) | 73 (40.6) |  |
| Perceived Income |  |  |  |  |  |  | X^2^ = 5.4  ΔX^2^ =85.1 |
| My income is less than my expenses | 22 (21.0) | 6 (6.7) | 17 (16.2) | 5 (5.6) | 39 (18.6) | 11 (6.1) |  |
| My income equals my expenses | 32 (30.5) | 22 (24.4) | 20 (19.0) | 18 (20.0) | 52 (24.8) | 40 (22.2) |  |
| My income more than my expenses | 51 (48.6) | 62 (68.9) | 68 (64.8) | 67 (74.4) | 119 (56.7) | 129 (71.7) |  |
| Parental Status |  |  |  |  |  |  | X^2^ = .00  ΔX^2^ =25.7 |
| No | 8 (7.6) | 2 (2.2) | 8 (7.6) | 2 (2.2) | 16 (7.6) | 4 (2.2) |  |
| Yes | 97 (92.4) | 88 (97.8) | 97 (92.4) | 88 (97.8) | 194 (92.4) | 176 (97.8) |  |
| Living Alone |  |  |  |  |  |  | X^2^ = .82  ΔX^2^ =19.38 |
| No | 65 (61.9) | 80 (88.9) | 57 (54.3) | 80 (88.9) | 122 (58.1) | 160 (88.9) |  |
| Yes | 40 (38.1) | 10 (11.1) | 48 (45.7) | 10 (11.1) | 88 (41.9) | 20 (11.1) |  |
| Descriptive statistics are shown as the number together with % in parentheses. *p< .05; **p< .01; ***p< .001 | | | | | | | |

**Table AF3.** Sample Description by gender and ethnicity **(After Manual Matching)**

|  | **Turkish Sample**  **(N=195)** | | **German Sample**  **(N=195)** | | **Total Sample**  **(N=390)** | | **Test of differences between Turkish and German** |
| --- | --- | --- | --- | --- | --- | --- | --- |
| Measure | **Female**  **(N=105)** | **Male**  **(N=90)** | **Female**  **(N=105)** | **Male**  **(N=90)** | **Female (N=210)** | **Male (N=180)** |  |
|  | N (%) | N (%) | N (%) | N (%) | N (%) | N (%) | Δ Subsamples |
| Age Group |  |  |  |  |  |  | X^2^ = .00  ΔX^2^ =87.2 |
| 75-79 | 77 (73.3) | 57 (63.3) | 77 (73.3) | 57 (63.3) | 154 (73.3) | 114 (63.3) |  |
| 80+ | 28 (26.7) | 33 (36.7) | 28 (26.7) | 33 (36.7) | 56 (26.7) | 66 (36.7) |  |
| Relationship Status |  |  |  |  |  |  | X^2^ = .01  ΔX^2^ =6.9 |
| Have not partner | 53 (50.5) | 14 (15.6) | 54 (51.4) | 14 (15.6) | 107 (51.0) | 28 (15.6) |  |
| Have a partner | 52 (49.5) | 76 (84.4) | 51 (48.6) | 76 (84.4) | 103 (49.0) | 152 (84.4) |  |
| Level of Education |  |  |  |  |  |  | X^2^ = 10.8*  ΔX^2^ =71.6 |
| Unable to read and write | 20 (19.0) | 1 (1.1) | 4 (3.8) | 4 (4.4) | 24 (11.4) | 5 (2.8) |  |
| Elementary school | 64 (61.0) | 56 (62.2) | 61 (58.1) | 50 (55.6) | 125 (59.5) | 106 (58.9) |  |
| Secondary school | 11 (10.5) | 12 (13.3) | 27 (25.7) | 12 (13.3) | 38 (18.1) | 24 (13.3) |  |
| (technical) high school and over | 10 (9.5) | 21 (23.3) | 13 (12.4) | 24 (26.7) | 23 (11.0) | 45 (25.0) |  |
| Household net income |  |  |  |  |  |  | X^2^ = 64.1***  ΔX^2^ =233.0 |
| Less than € 1000 | 31 (29.5) | 14 (15.6) | 13 (12.4) | 4 (4.4) | 44 (21.0) | 18 (10.0) |  |
| €1001 - €1500 | 45 (42.9) | 38 (42.2) | 29 (27.6) | 20 (22.2) | 74 (35.2) | 58 (32.2) |  |
| €1501 - €2000 | 23 (21.9) | 30 (33.3) | 30 (28.6) | 23 (25.6) | 53 (25.2) | 53 (29.4) |  |
| More than €2001 | 6 (5.7) | 8 (8.9) | 33 (31.4) | 43 (47.8) | 39 (18.6) | 51 (28.3) |  |
| Perceived Income |  |  |  |  |  |  | X^2^ = 3.3  ΔX^2^ =87.2 |
| My income is less than my expenses | 22 (21.0) | 6 (6.7) | 17 (16.2) | 7 (7.8) | 39 (18.6) | 13 (7.2) |  |
| My income equals my expenses | 32 (30.5) | 22 (24.4) | 21 (20.0) | 20 (22.2) | 53 (25.2) | 42 (23.3) |  |
| My income more than my expenses | 51 (48.6) | 62 (68.9) | 67 (63.8) | 63 (70.0) | 118 (56.2) | 125 (69.4) |  |
| Parental Status |  |  |  |  |  |  | X^2^ = .00  ΔX^2^ =25.7 |
| No | 8 (7.6) | 2 (2.2) | 8 (7.6) | 2 (2.2) | 16 (7.6) | 4 (2.2) |  |
| Yes | 97 (92.4) | 88 (97.8) | 97 (92.4) | 88 (97.8) | 194 (92.4) | 176 (97.8) |  |
| Living Alone |  |  |  |  |  |  | X^2^ = 1.0  ΔX^2^ =19.2 |
| No | 65 (61.9) | 80 (88.9) | 59 (56.2) | 77 (85.6) | 124 (59.0) | 157 (87.2) |  |
| Yes | 40 (38.1) | 10 (11.1) | 46 (43.8) | 13 (14.4) | 86 (41.0) | 23 (12.8) |  |
| Descriptive statistics are shown as the number together with % in parentheses. *p< .05; **p< .01; ***p< .001 | | | | | | | |

**Item-total correlations analysis of Beck Depression Inventory**

**Table AF4.** Items of K5-Beck Depression Inventory

|  | 1 | 2 | 3 | 4 | 5 | 6 |
| --- | --- | --- | --- | --- | --- | --- |
| 1. I feel sad. | - |  |  |  |  |  |
| 2. I am disappointed in myself. | .724** | - |  |  |  |  |
| 3. I am tired and listless. | .713** | .673** | - |  |  |  |
| 4. It is difficult for me to enjoy something. | .702** | .680** | .725** | - |  |  |
| 5. I put off making decisions. | .443** | .485** | .587** | .537** | - |  |
| 6. Beck Depression Inventory (Item-total correlation) | .870** | .848** | .880** | .873** | .714** | - |
| *p< .05; **p< .01; ***p< .001; N=195 | | | | | | |

Depression of Turkish older adults were evaluated with 5 items of the Beck Depression Inventory in this study. Participated rated each of the 5 items in response to the question: *“How often did you experience each mood or view during the past 1 week?”. A* five-point Likert Scale was used with 1= “*never*” and 5= “*almost always*”.

To make certain that items expressed culturally contoured meanings, item-total correlations analysis were performed for the 5 items. A high correlation coefficient for each item indicates that the item measures the intended behavior effectively and adequately (Hair, 2009). The item-total correlations for BDI-V (*n=*195) ranged from 0.71–0.88 for all items, expressing that the items were homogeneous and had the best coefficients. Additionally, the internal consistency of the Beck Depression Inventory was addressed using the Cronbach α coefficient. The reliability coefficients were α= .89 for Turkish sample and α= .80 for German sample in the current study. This finding is similar to the original German version of BDI-V with α=.90 (Schmitt & Maes, 2000), and also similar to one other study including older German adults with α= .84 (Rohr et al., 2017). This suggests that the reliability of the Turkish version of the scale is compatible with the original form.

**Additional references**

Hair Jr, J. F. (2009). Multivariate Data Analysis Joseph F. Hair Jr. William C. Black Barry J. Babin Rolph E. Anderson Seventh Edition. ss.161.

Rohr, M. K., John, D. T., Fung, H. H., & Lang, F. R. (2017). A three-component model of future time perspective across adulthood. *Psychology and Aging, 32*(7), 597–607. <http://doi.org/10.1037/pag0000191>.

Schmitt, M., & Maes, J. (2000). Vorschlag zur Vereinfachung des Beck-Depressions-Inventars (BDI). *Diagnostica, 46*(1), 38–46. Hogrefe-Verlag Göttingen.

**Table AF5.** Bivariate Correlation by gender among Turkish sample

| Women  Men | M | SD | 1 | 2 | 3 | 4 | 5 | 6 | 7 |  |
| --- | --- | --- | --- | --- | --- | --- | --- | --- | --- | --- |
| M |  |  | 2.90 | 78.61 | 2.66 | 3.84 | 2.03 | 2.25 | 61.68 |  |
| SD |  |  | 1.1 | 3.5 | 1.2 | .68 | .79 | .74 | 26.8 |  |
| 1.Depression | 2.68 | 1.2 | - | .041 | **.313^**^** | **-.338^***^** | **-.434^***^** | **-.339^***^** | **-.461^***^** |  |
| 2.Age | 79.50 | 3.8 | .008 | - | .105 | -.144 | .129 | **-.203^*^** | -.094 |  |
| 3. Received Support | 2.26 | 1.1 | **.302^**^** | -.033 | - | .124 | -.163 | -.116 | **-.200^*^** |  |
| 4.Perceived Support | 3.81 | .65 | **-.244^*^** | **-.361^***^** | **.236^*^** | - | .170 | **.459^***^** | **.242^*^** |  |
| 5.Family Satisfaction | 1.97 | .73 | **-.327^**^** | .010 | .115 | **.345^***^** | - | **.348^***^** | .102 |  |
| 6.Friend Satisfaction | 1.94 | .72 | **-.262^*^** | -.140 | .175 | **.440^***^** | **.445^***^** | - | .102 |  |
| 7.Subjective Health | 65.57 | 23.2 | **-.449^***^** | -.079 | -.071 | **.276^**^** | **.286^**^** | .165 | - |  |
| Coefficients printed in bold are significant (* *p* < .05; ** *p* < .01; *** *p* < .001); N=105 for women and N=90 for men.  The below of the diagonal represents men and the above represents women. | | | | | | | | | | |

**Table AF6.** Bivariate Correlation by gender among German sample

| Women  Men | M | SD | 1 | 2 | 3 | 4 | 5 | 6 | 7 |
| --- | --- | --- | --- | --- | --- | --- | --- | --- | --- |
| M |  |  | 2.20 | 78.63 | 2.69 | 4.10 | 2.44 | 2.10 | 68.67 |
| SD |  |  | .85 | 3.5 | .75 | .72 | .66 | .59 | 19.3 |
| 1.Depression | 1.97 | .63 | - | **.371^***^** | **.345^***^** | -.188 | .016 | -.186 | **-.517^***^** |
| 2.Age | 79.46 | 3.6 | -.102 | - | **.194^*^** | -.010 | .083 | -.151 | **-.261^**^** |
| 3.Received Support | 2.41 | .73 | -.138 | .109 | - | .178 | .054 | .003 | -.189 |
| 4.Perceived Support | 4.02 | .82 | **-.458^***^** | -.053 | .**393^***^** | - | **.317^***^** | **.291^**^** | .152 |
| 5.Family Satisfaction | 2.43 | .67 | **-.334^**^** | -.064 | .199 | **.478^***^** | - | **.340^***^** | .132 |
| 6.Friend Satisfaction | 1.98 | .64 | **-.335^**^** | -.045 | **.288^**^** | **.524^***^** | **.418^***^** | - | .167 |
| 7.Subjective Health | 73.37 | 16.8 | -.029 | .005 | **-.245^*^** | -.038 | .073 | -.019 | - |
| Coefficients printed in bold are significant (* *p* < .05; ** *p* < .01; *** *p* < .001); N=105 for women and N=90 for men.  The below of the diagonal represents men and the above represents women. | | | | | | | | | |

**Acknowledgements and Author Note.**

Correspondence concerning this article should be addressed to N. Tugba Bahar or Frieder R. Lang, Institute of Psychogerontology, Universität Erlangen-Nürnberg, Kobergerstr. 62, 90408 Nuremberg, Germany. E-mail: [tugba.bahar@fau.de](mailto:tugba.bahar@fau.de) or to [frieder.lang@fau.de](mailto:frieder.lang@fau.de). The research was supported by the Theo and Friedl Schöller Foundation Nurnberg, Germany to Frieder R. Lang. N. Tugba Bahar was supported with the Study Abroad Postgraduate Education Scholarship (YLSY) awarded by the Republic of Türkiye Ministry of National Education.
